# Youth Advocacy as a Tool for Environmental and Policy Changes That Support Physical Activity and Nutrition: An Evaluation Study in San Diego County

**DOI:** 10.5888/pcd11.130321

**Published:** 2014-03-27

**Authors:** Leslie S. Linton, Christine C. Edwards, Susan I. Woodruff, Rachel A. Millstein, Cheryl Moder

**Affiliations:** Author Affiliations: Christine C. Edwards, Health Policy Consulting Group, San Diego, California; Susan I. Woodruff, San Diego State University School of Social Work, San Diego, California; Rachel A. Millstein, San Diego State University/University of California at San Diego Joint Doctoral Program in Clinical Psychology, San Diego, California; Cheryl Moder, Community Health Improvement Partners, San Diego County Childhood Obesity Initiative.

## Abstract

**Background:**

As evidence grows about the benefits of policy and environmental changes to support active living and healthy eating, effective tools for implementing change must be developed. Youth advocacy, a successful strategy in the field of tobacco control, should be evaluated for its potential in the field of obesity prevention.

**Community Context:**

San Diego State University collaborated with the San Diego County Childhood Obesity Initiative to evaluate Youth Engagement and Action for Health! (YEAH!), a youth advocacy project to engage youth and adult mentors in advocating for neighborhood improvements in physical activity and healthy eating opportunities. Study objectives included documenting group process and success of groups in engaging in community advocacy with decision makers.

**Methods:**

In 2011 and 2012, YEAH! group leaders were recruited from the San Diego County Childhood Obesity Initiative’s half-day train-the-trainer seminars for adult leaders. Evaluators collected baseline and postproject survey data from youth participants and adult group leaders and interviewed decision makers.

**Outcomes:**

Of the 21 groups formed, 20 completed the evaluation, conducted community assessments, and advocated with decision makers. Various types of decision makers were engaged, including school principals, food service personnel, city council members, and parks and recreation officials. Eleven groups reported change(s) implemented as a result of their advocacy, 4 groups reported changes pending, and 5 groups reported no change as a result of their efforts.

**Interpretation:**

Even a brief training session, paired with a practical manual, technical assistance, and commitment of adult leaders and youth may successfully engage decision makers and, ultimately, bring about change.

## Background

As evidence grows about the benefits of policy and environmental changes to support active living and healthy eating (1), practitioners need evidence of successful policy implementation strategies. This is particularly true in at-risk communities where environmental inequities are associated with low levels of physical activity and healthy eating (2). Advocacy is a community-based strategy of education, communication, and leadership skills for persuading others about a point of view. Advocacy for physical activity environment and healthy eating policy changes is an understudied approach (3).

The success of youth advocacy in reducing youth tobacco use provides useful program and evaluation models (4). Youth advocacy has strengthened clean indoor air laws, established smoke-free campuses, and led to zoning changes addressing industry advertising in low-income neighborhoods (5). There are several research-based examples of successful tobacco control youth advocacy programs. One set of studies (American Legacy Foundation’s Statewide Youth Movement Against Tobacco Use [SYMATU]) examined the conceptual and practical factors involved in youth empowerment and advocacy programs in tobacco control. This conceptual framework included predisposing youth characteristics, collective participation, group structure, adult and institutional involvement, and group climate; outcomes were at the individual, group, community, and society-wide levels (6). This study found that counter-industry and interpersonal confidence, perceived sociopolitical control, participatory competence, knowledge of resources, assertiveness, and advocacy were associated with youth empowerment (6).

Theory and practice suggest that youth advocacy for obesity prevention is a promising strategy (1). Youth advocacy has potential for impact. Decision makers may be less cynical about youth’s motives and less prone to reject their requests. Young people possess enthusiasm and optimism that can be harnessed to promote community-based changes (1). Additionally, the prevalence of obesity is highest among low-income groups and some racial/ethnic racial minority groups (7), and efforts to reduce health disparities could benefit from emphasizing youth advocacy (1). We describe a local obesity initiative’s program for training and supporting adult leaders of youth advocacy groups pursuing obesity-related policy and environmental changes. We describe the program’s evaluation, present results for 21 youth advocacy groups, and discuss implications for practitioners and researchers.

## Community Context

With a population of more than 3 million, San Diego County is the fifth most populous county in the United States (8). During the last decade, the Hispanic population in San Diego County has grown by a third, to 32% of the population (9). Public school enrollment for 2011–2012 reflects the region’s diversity: 46.5%, Hispanic; 32.4%, white; 5.8%, black; 5.8%, Asian; 4.3%, Filipino; and 3.2%, “2 or more races” (10). English learners are 23% of the public school population, and 48% qualify for free or reduced-price lunches (10).

In 2011, more than 33% of those aged 12 to 17 years in Southern California were overweight or obese (11). Fewer than 25% of those aged 5 to 17 years engaged in regular weekly physical activity (at least 1 hour daily, excluding physical education) (11). In 2011–2012, more than 40% of children in grades 5, 7, and 9 in San Diego County did not meet the Healthy Fitness Zone for body composition, a criterion-referenced standard established by the Cooper Institute (12). Among racial/ethnic groups, disparities exist: among fifth-graders, a greater percentage of Hispanic students (53%) than white students (32%) did not meet Healthy Fitness Zone standards for body composition (13).

The San Diego County Childhood Obesity Initiative (COI), a program of the nonprofit Community Health Improvement Partners, is a public–private partnership with the mission of reducing and preventing childhood obesity through policy, systems, and environmental change. The COI is structured on 7 domains: government, health care, school and after-school, early childhood, community, media, and businesses. More than 100 community partners participate. COI is governed by a leadership council, which is co-chaired by 2 private pediatricians and San Diego County’s Public Health Officer (14).

Youth Engagement and Action for Health! (YEAH!) is a youth advocacy and community-empowerment program that engages local youth and adult mentors in advocating for neighborhood improvements in physical activity opportunities and access to healthy foods. YEAH! empowers youth to assess their environments, prioritize problems based on those assessments, and develop and implement an action plan to advocate with decision makers for changes. The YEAH! curriculum and training for adult leaders grew from the County of San Diego Health and Human Services Agency’s pilot projects and is an outgrowth of the California Department of Health Service’s (CDHS) Network for a Healthy California’s project called Communities of Excellence in Nutrition, Physical Activity and Obesity Prevention (CX^3^) and CDHS’s Tobacco Control Section Communities of Excellence planning framework. In addition to implementing YEAH! trainings, COI provides technical assistance to groups implementing YEAH!, including in-person, e-mail, and telephone meetings with group leaders.

San Diego State University collaborated with COI to implement an evaluation of the adult-led groups of youth pursuing YEAH! projects after adult leader training seminars. We report on 2 objectives of our evaluation: 1) to assess the extent to which groups succeeded in engaging in community advocacy with decision makers and 2) to note preliminary successes in securing policy and environmental change as a result of group efforts. Analysis of data on an additional objective, to understand the effect on youth participating in advocacy, is in process.

## Methods

### Intervention

Biannually, COI holds half-day train-the-trainer seminars for adult leaders of youth groups that have an interest in working on healthy eating or active living community advocacy projects. Groups may form as part of school classes or groups, community organizations, after-school programs, religious organizations, and teen centers. The YEAH! manual, available to trainees and to others on request to COI, includes instructions on recruiting youth, gathering resources, conducting community assessments of modifiable environmental factors that affect health (parks, fast food outlets, schools, stores, and outdoor advertising), identifying decision makers, and learning to advocate. Groups choose 1 or more assessments to conduct, and on the basis of assessment data, prioritize problems and develop advocacy plans to address 1 or more issues. Youth typically use the assessments, together with photographs or video, to document potential neighborhood or school environmental problems. On completion of their assessments, youth compile their findings into an advocacy presentation, identify relevant decision makers, and deliver their presentation in person, in writing, or through other modes.

The suggested timeline for YEAH! varies with the complexity of project chosen; the manual suggests 4 to 6 months. All groups are run independently, and group leaders have discretion to determine the duration of the project and the frequency and agenda of meetings. During training, group leaders were encouraged to seek technical assistance from the COI as needed. Technical assistance furnished by COI included locating specialized assessment forms and other materials, identifying outside experts to render advice, assisting with identifying appropriate decision makers, and developing advocacy strategies. A brief video (www.yeahsandiego.org) made by several groups formed during the evaluation summarizes the YEAH! process.

### Evaluation

**Figure 1:**
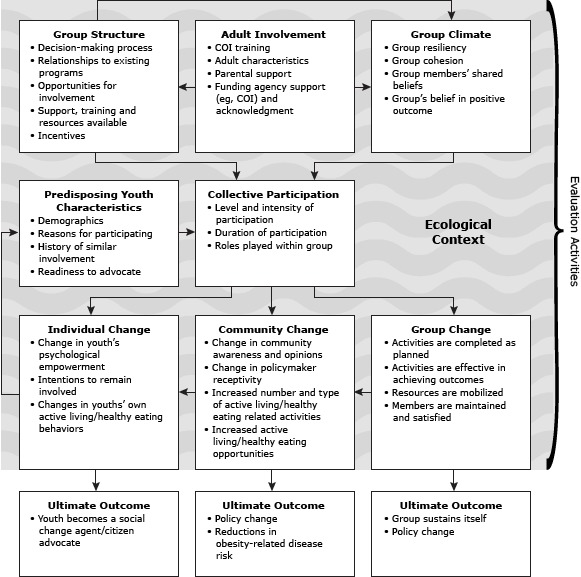
Youth-engagement framework based on a framework for an evaluation of the Statewide Youth Movement Against Tobacco Use, a multi-state youth advocacy program in tobacco control (6). Abbreviation: COI, San Diego County Childhood Obesity Initiative.

The evaluation (e-YEAH!) was funded through a grant from the Robert Wood Johnson Foundation and administered through its Active Living Research Program. The conceptual model (Figure 1) drew on the framework for an evaluation of SYMATU (6).

**Table:** 


Group Structure (Evaluation Activities)
— Decision-making process
— Relationships to existing programs
— Opportunities for involvement
— Support, training & resources available
— Incentives
Predisposing Youth Characteristics (Evaluation Activities) (Ecological Context)
— Demographics
— Reasons for participating
— History of similar involvement
— Readiness to advocate
Individual Change (Evaluation Activities)
— Change in youth's psychological empowerment
— Intentions to remain involved
— Changes in youths' own active living/healthy eating behaviors
Ultimate Outcome
— Youth becomes a social change agent/citizen advocate
Adult Involvement (Evaluation Activities)
— COI training
— Adult characteristics
— Parental support
— Funding agency support (eg, COI) and acknowledgment
Collective Participation (Evaluation Activities) (Ecological Context)
— Level and intensity of participation
— Duration of participation
— Roles played within group
Community Change (Evaluation Activities)
— Change in community awareness and opinions
— Change in policymaker receptivity
— Increased number and type of active living/healthy eating related activities
— Increased active living/healthy eating opportunities
Ultimate Outcome
— Policy change
— Reductions in obesity-related disease risk
Group Climate (Evaluation Activities)
— Group resiliency
— Group cohesion
— Group members' shared beliefs
— Group's belief in positive outcome
Group Change (Evaluation Activities)
— Activities are completed as planned
— Activities are effective in achieving outcomes
— Resources are mobilized
— Members are maintained and satisfied
Ultimate Outcome
— Group sustains itself
— Policy change

The model recognizes multiple levels of influence leading to outcomes, including predisposing characteristics of participants, extent of participation, external support, and group climate, all taking place in various ecological contexts.

**Figure 2:**
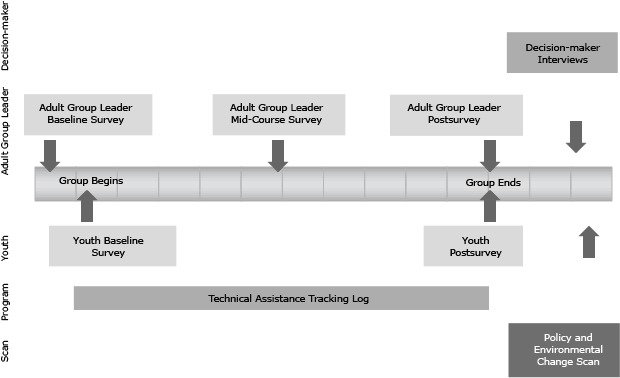
Timetable for San Diego County YEAH! Evaluation, 2011–2013.

To measure influences at multiple levels, evaluators collected qualitative and quantitative data through surveys administered to youth and adult group leaders at baseline and after completion of each group’s project. Using a standardized interview protocol, evaluators also interviewed decision makers to whom YEAH! groups had made advocacy presentations. Decision makers were interviewed between 2 weeks and 6 months after the advocacy presentations (Figure 2). Pre- and post-surveys of youth assessed attitudinal and behavioral changes, as well as perceptions of control, self-efficacy, and readiness to act as social-change advocates. Baseline, midcourse, and postproject online surveys of adult leaders assessed their characteristics, group structure and dynamics, barriers to success, process information, and technical assistance needs. Interviews with decision makers assessed perceptions of interactions with youth advocates. This article reports on data from adult leader surveys, decision-maker interviews, and baseline surveys of youth demographics.

Adult group leaders in the evaluation study were recruited through the COI training seminars between January 2011 and November 2012. Group leaders who consented received a $150 cash incentive to spend on group-related costs. Youth members of YEAH! groups were recruited for the evaluation study during their first group meeting. Parent consent forms were available in English and Spanish; all surveys were conducted in English. Youth with signed consent and assent forms were asked to complete a written baseline survey and a postproject survey. Gift cards from Subway or Jamba Juice were given to youth and adults who completed the baseline ($10) and postproject survey ($15). San Diego State University’s institutional review board approved the study.

## Outcome

At various times during the 2-year evaluation period, 21 groups were formed, and all consented to participate in the evaluation: 20 groups and 136 young participants completed the baseline surveys. We did not collect baseline data from 1 group because the evaluation team did not learn of the group until it was well under way. We estimate that at least 90% of youth participants participated in the baseline survey. There were 52 adult group leaders for the 21 groups; 47 leaders representing 20 groups completed the baseline surveys; and 44 leaders representing 18 groups completed the postproject surveys. Most of the youth participants were girls (73%), and participants were racially/ethnically diverse: 36% were Hispanic; 23% were non-Hispanic black; and 22% were Asian, Pacific Islander, or Native Hawaiian (Table 1). Leaders ranged in age from 22 to 64 (Table 2), and about two-thirds were non-Hispanic white. Groups had 1 to 12 adult leaders; all groups except one had from 1 to 5 adult leaders.

**Table 1:** Self-Reported Characteristics of Participants at Baseline (N = 136), San Diego County YEAH! Projects, 2011–2013

Characteristic	No. of Respondents	Value
**Sex, %**		
Female	134	73
Male		27
**Race/ethnicity,^a^ %**		
Hispanic	132	36
Black non-Hispanic		23
Asian/Pacific Islander/Native Hawaiian		22
White non-Hispanic		13
Other		15
**Age, median (range), y**	136	15 (9–22)
**Earn better grades than average, %**	133	58
**Some previous advocacy activity, %**	136	74
**No. of days per week physically active, mean(SD)**	136	3.7 (2.2)
**Times per week eat fast food, mean (SD)**	124	1.6 (1.7)

Abbreviations: YEAH, Youth Engagement and Action for Health; SD, standard deviation.

a Respondents could choose more than 1 category, so percentages do not add to 100.

**Table 2:** Characteristics of Groups (n = 21) and Leaders (n = 47), San Diego County YEAH! Projects, 2011–2013

Characteristic (no. of groups)	Value
**Setting (n = 21)**	
High school, n	6
Middle school, n	6
Community center, n	8
Church, n	1
**Duration of project^a^ (n = 18)**	
No. of sessions per group, mean	9
No. of weeks from start to completion, mean	10
No. of hours met per group, mean	19
**Size and retention^a^ (n = 18)**	
No. of participants per group at project completion, mean	7
Participants engaged through project completion, %	73
**Leaders (n = 18)**	
No. of leaders per group,^a^ range	1–12
Age of leaders,^a^ range, y	22–64
No. of hours per leader devoted to YEAH!,^a^ mean	31
**Groups that had at least 1 paid leader^b^ (n = 20), n**	14
**Leader experience among groups^b^ (n = 20)**	
Groups had worked with leader previously, n	7
Groups with at least 1 leader with experience in physical activity and nutrition, n	17
Groups with at least 1 leader with experience in fields of policy, education, or neighborhood design, n	16
Groups with at least 1 leader with previous experience in youth advocacy, n	9
**Use of external consultants^a^ (n = 18)**	
Groups consulted with any outside expert, n	14
Groups consulted with San Diego County Childhood Obesity Initiative, n	12

Abbreviation: YEAH, Youth Engagement and Action for Health.

a Data source: postproject survey of 44 group leaders.

b Data source: baseline survey of 47 group leaders.

Groups formed in 3 settings: 6 in high schools, 6 in middle schools, and 8 in community centers (Table 2). Groups met for an average of 9 sessions during a 10-week period. Group leaders devoted an average of 31 hours to the project; 70% of the leaders were compensated, either as a part of paid work duties or through a small stipend. Most (n = 14) groups consulted with an outside expert during their YEAH! project. Among participants who began the project, 73% completed it.

Twenty groups engaged in advocacy (Table 3). Most (n = 13) conducted school assessments, 5 assessed parks, and 2 assessed outdoor advertising. Fast food, store, and street advertising assessments were each conducted by 1 group. On the basis of these assessments, 8 groups identified issues with playgrounds, parks, and recreation facilities, and 5 groups identified issues relating to school food. All 20 groups engaged in advocacy with decision makers; 19 groups completed in-person presentations or meetings. Eleven groups reported a change implemented as a result of their advocacy, 4 groups reported changes pending, and 5 groups saw no change as a result of their efforts (15). Advocacy resulted in changes such as the addition of a salad bar at a high school, the addition of exterior lighting at a community center so youth could walk to and from the center at night, and the addition of female-only swim time at a YMCA, facilitating participation by young Muslim women.

**Table 3:** Groups Engaged in Advocacy Activities, San Diego County YEAH! Projects, 2011–2013

Activity	No. of Groups
**Conducted Community Assessment (n = 20)**	
**Type of assessment conducted^a^^, ^ b**	
Schools	13
Parks	5
Fast food	1
Outdoor advertising	2
Store	1
Street	1
**Issues identified for which improvement was sought^b^^, ^ c**	
Playgrounds, parks, recreation facilities	8
School food: cafeteria and vending machines	5
School drinking water	2
Crosswalks and sidewalks	2
School fundraising practices	1
Local stores: availability of fresh produce	1
Outdoor advertising	1
School health education policy	1
**Engaged in Community Advocacy (n = 20)**	
**Engagement process^a^^, ^ b**	
In-person presentations or meetings	19
Letters, e-mails, or telephone calls	7
Worked with media	1
**Decision makers engaged^a^^, ^ b**	
School principal	13
Food service personnel	5
School board	3
Parks or recreation personnel	3
City council	3
City or county planning organizations	3
Store or business owners	1
Parent–teacher association	1
Police	1
**Policy or environmental outcomes^c^**	
Changes implemented	11
Changes pending	4
No changes identified	5
No information	1

Abbreviation: YEAH, Youth Engagement and Action for Health.

a Data source: postproject survey of group leaders representing 18 groups and 2 interviews of leaders representing 2 groups.

b Respondents could choose more than 1 category.

c Data source: postproject survey of group leaders representing 18 groups and 14 decision-maker interviews representing 13 groups.

## Interpretation

This study presents a theory-based evaluation of advocacy, a novel approach to community youth obesity prevention. The evaluation informed COI’s continuation of YEAH! and may assist other communities considering youth advocacy strategies.

The YEAH! groups we studied were generally of relatively short duration, with groups meeting for an average of 9 sessions totaling an average of 19 hours per group during 10 weeks. For some groups, according to qualitative data collected from group leaders after project completion, the duration was constrained by resources and time. The short duration may also reflect a lack of experience in youth advocacy; only 9 groups had leaders experienced in youth advocacy (Table 2), so many leaders may not have understood the advantage of allowing more time to complete the process. Implementing YEAH! projects in a short time may also have resulted in leaders guiding youth to investigate issues that could be addressed in a short time and away from those that would need more time. Even though the youth investigators may have discovered serious built-environment issues worthy of their attention during the assessment phase of their projects (eg, need for sidewalks outside a school), other issues (eg, school menu) may have seemed more within reach.

Fourteen groups accessed technical assistance from outside experts, and 12 groups consulted with COI. When evaluators contacted groups mid-project, leaders often explained that their groups had completed assessments but were not clear about the next steps in engaging decision makers. More proactive technical assistance may be indicated so that leaders benefit from guidance about political decision-making structures (eg, identifying who decides whether or not a school can get a joint-use field agreement for an adjoining city-owned property).

Among the 12 groups that met at public high schools or middle schools, almost all met at schools with high rates of free or reduced-price lunches (range, 34%–92%; mean, 76%). Groups that met at community centers met at after-school centers for children of Navy personnel (n = 3), affordable housing resident groups (n = 3), a community-based organization for East African young women (n = 1) and a job training program for youth on probation (n = 1). The successful implementation of programs in these settings is notable, particularly given the higher prevalence of childhood obesity in underresourced communities. Several groups were based in high schools as part of health science pipeline programs to educate and encourage youth to pursue health-related careers. This setting provides an opportunity to train a new generation of health professionals to be current and future advocates for policies supporting wellness. Institutionalizing YEAH! groups in any of these settings would leverage leader experience, allow youth to mentor each other, and offer the continuity needed to address issues requiring sustained advocacy.

Following completion of the evaluation, COI modified the YEAH! training seminars and manual to incorporate the above-mentioned video, more assessment instruments, expanded resources and advocacy planning information, and case studies of successful groups. In 2013, working through its community domain, COI fielded a request for proposals to youth-serving organizations interested in developing YEAH! groups. COI will provide a stipend of $800 to each of the 4 groups selected to participate, as well as technical assistance from a COI advisor who trains leaders, offers individual consultations, and coordinates networking meetings of active group leaders. COI continues to seek funding for the YEAH! coordinator, and future plans are to institutionalize YEAH! in youth-serving organizations such as schools with health-science programs.

Key elements of the YEAH! program included 1) close partnerships with organizations serving youth, 2) committed adult leaders trained in the importance of policy and environmental change, and 3) ongoing support to adult leaders from experts with the skills to identify decision makers and facilitate advocacy connections. A community initiative like COI, with existing community partnerships, as well as experienced staff trained in local political process and policy and environmental approaches to obesity issues, was an efficient means to provide the infrastructure and continuity to support the program.

We are currently analyzing short-term positive changes among the participating youth based on pre- and post-survey data (16). Long-term follow-up of youth, as well as follow-up of their group leaders, would document the benefits of participation. Longer-term research could also document policy and environmental changes following advocacy and maintenance of changes over time. This information would help guide resource allocation for future youth advocacy efforts.

At the outset of the evaluation, it was unclear if a half-day YEAH! leader training seminar would be adequate to provide skills and information to leaders to engage groups of youth. This evaluation provides evidence that the YEAH! model of providing a brief training, paired with a practical manual and ongoing support for adult mentors, was successful in engaging youth to conduct assessments and advocacy with decision makers in a variety of school and community settings, including groups located in underresourced schools and communities. Ongoing technical support to leaders was important in bridging the process between initial neighborhood assessment and final advocacy with decision makers. As the field accumulates evidence about which policy and environmental changes will best support physical activity and nutrition, it should continue to explore how communities can effectively implement these changes. Next steps in exploring youth advocacy as a policy-change strategy should include exploration of the extent and maintenance of changes secured through advocacy, the potential for institutionalization of youth advocacy in youth-serving organizations, and long-term effects of participation on youth and adult participants.
